# Changes in pediatric oncology nurses’ knowledge and observed mucormycosis-prevention practices following an educational programme: a one-group pretest-posttest study

**DOI:** 10.1186/s12912-026-05382-5

**Published:** 2026-10-12

**Authors:** Amira El-Sayed, Sabah Alsharkawy, Eman Abdelfattah Hassan, Hoda Wahid, Nema Ragab, Hagar Abd El-Hamid

**Affiliations:** 1 Faculty of Nursing, October 6 University, Giza, Egypt; 2 Faculty of Nursing, Ain Shams University, Cairo, Egypt; 3 Faculty of Nursing, Cairo University, Cairo, Egypt; 4 Faculty of Nursing, Misr University for Science and Technology, Giza, Egypt

**Keywords:** Mucormycosis, Child, Nursing care, Chemotherapy, Practice

## Abstract

**Background:**

Mucormycosis is a life-threatening fungal infection increasingly affecting immunocompromised individuals, including children receiving chemotherapy. Its rising prevalence and severe outcomes highlight the importance of prevention. Nurses play a pivotal role, requiring improved knowledge and strict adherence to infection control practices.

**Aim:**

This study aimed to evaluate the changes in pediatric oncology nurses’ knowledge and observed mucormycosis-prevention practices following an educational programme.

**Methods:**

A quasi-experimental design with pre- and posttest assessments were used. The study was conducted at the Children’s Cancer Hospital Egypt (CCHE) 57,357 and included 81 nurses. Data were collected using a structured questionnaire to assess characteristics and knowledge, along with an observational checklist designed to evaluate nurses’ practices. The educational intervention consisted of three sessions covering mucormycosis risk factors, pathophysiology, and infection control measures.

**Results:**

The mean knowledge score increased from 5.82 ± 0.91 to 12.1 ± 0.76 (*p* < 0.001), and the mean practice score increased from 3.86 ± 1.13 to 7.58 ± 1.02 (*p* < 0.001). A positive correlation was found between knowledge and practice scores in both preintervention (*r* = 0.348, *p* = 0.001) and postintervention (*r* = 0.382, *p* < 0.001). Knowledge and observed practice scores increased significantly following the educational sessions.

**Conclusion:**

Structured educational sessions were associated with significant short-term improvements in nurses’ knowledge and observed mucormycosis-prevention practices. As patient outcomes and mucormycosis incidence were not measured, these findings should not be interpreted as evidence of reduced infection rates. Continuous professional development and targeted training are important for strengthening infection prevention knowledge and practices among pediatric oncology nurses.

**Clinical trial number:**

Not applicable.

## Introduction

Mucormycosis is a rare filamentous fungal infection caused by fungi belonging to the order Mucorales [1]. It is also known as zygomycosis [2, 3] and is commonly known as black fungus [4]. It primarily affects individuals with weakened immune systems, such as those with diabetes, severe burns, blood disorders, kidney illness, sepsis, or extreme dehydration [5]. According to the Leading International Fungal Education (LIFE) Organization, mucormycosis is becoming increasingly prevalent in children, where it is associated with significant morbidity and mortality rates [6]. Globally, the prevalence of mucormycosis is estimated to be around 10,000 cases per year [1]. Invasive mucormycosis is most reported in India (55.3%) and the Middle East and North Africa (MENA) region, particularly in Egypt (17.8%), Iran (9.9%), and Turkey (6.3%) [7].

A retrospective study conducted at the Children’s Cancer Hospital in Egypt from 2007 to 2017 documented 45 cases of proven mucormycosis among 13,735 pediatric oncology patients admitted to the hospital, equating to 3.2 cases per 1,000 pediatric cancer patient admissions [8]. Children at risk of mucormycosis often have conditions such as hematologic malignancies, leukemia, neutropenia, neonatal prematurity, malnutrition, or have undergone hematopoietic stem cell transplants (HSCT). The symptoms of mucormycosis vary depending on the location of the fungus in the body [9, 10]. The symptoms depend on the infection site and include localized soft tissue infections, sinopulmonary involvement, or disseminated infections affecting multiple non-contiguous body sites [8, 11].

The increasing prevalence of mucormycosis, particularly in pediatric oncology patients, highlights the urgency of effective infection prevention strategies. Given that pediatric oncology patients are especially vulnerable to infections, the role of healthcare providers in preventing these infections becomes critical [12]. Caring for pediatric patients undergoing chemotherapy is a complex and multidimensional process, making it essential for nurses to be thoroughly knowledgeable about handling and safety measures, as they are at risk of exposure to infections [13]. However, multiple studies have highlighted that effective infection prevention practices among nurses are often hindered by barriers such as a lack of knowledge, insufficient resources, and inadequate preventive guidelines [14]. Nurses, in particular, play an integral role in infection control practices, as they are often the primary caregivers and are responsible for administering chemotherapy, managing patient care, and ensuring adherence to infection prevention protocols [15–18].

Nurses occupy a pivotal position within the multidisciplinary healthcare team caring for pediatric oncology patients, extending far beyond the administration of chemotherapy. Their responsibilities encompass continuous bedside monitoring for early signs of infection, strict adherence to hand hygiene and aseptic technique during invasive procedures, environmental infection control measures, and patient and family education regarding infection prevention practices at home and in the hospital setting. Given the prolonged and repeated contact nurses have with immunocompromised children, they are uniquely positioned to detect subtle clinical changes that may indicate an emerging fungal infection, enabling earlier diagnosis and intervention. Consequently, the depth and accuracy of nurses’ knowledge, coupled with their consistent application of evidence-based preventive practices, directly influence infection-related morbidity and mortality in this vulnerable population [19, 20].

However, despite the availability of established guidelines, studies have shown that the implementation of infection control measures remains inconsistent. This is largely due to gaps in knowledge, lack of adequate resources, and challenges in adhering to best practices. As the frontline healthcare professionals in pediatric oncology settings, nurses must be equipped with the knowledge and skills to mitigate the risk of infections like mucormycosis. Educational interventions targeting nurses’ knowledge and practices are therefore essential in enhancing infection control and improving patient outcomes in these high-risk settings.

### Aim

The aim of this study is to evaluate changes following structured educational sessions on nurses’ knowledge and practices regarding the prevention of mucormycotic infection in children receiving chemotherapy.

### Objectives

1. To assess the baseline knowledge and practices of nurses related to the prevention of mucormycotic infection before the educational intervention.
2. To determine post-intervention changes following the educational sessions on nurses’ knowledge about mucormycosis, including its causes, risk factors, and prevention strategies.
3. To evaluate changes in nurses’ infection prevention practices post-intervention, particularly in pediatric oncology settings.
4. To identify the relationship between increased knowledge and improved infection prevention practices among nurses.

### Hypothesis

It is hypothesized that the educational sessions will lead to a significant improvement in nurses’ knowledge and practices, thereby promoting better infection prevention measures compared to pre-intervention levels.

## Materials and methods

### Study design

The study employed a quasi-experimental design with pre-and post-test assessments of a single group, without a control group, conducted between January 2026 and April 2026.

### Study setting

The study was conducted at the Children’s Cancer Hospital Egypt (CCHE) 57357, located in Cairo, Egypt. CCHE 57357 is one of the world’s leading hospitals specializing in pediatric cancer care, providing a comprehensive range of services for children with cancer. The hospital, with its 350 beds, is divided among intensive care units (ICUs), inpatient wards, and outpatient clinics.

### Study subjects

A total population sampling (census) approach was used. All nurses actively involved in the care of children receiving chemotherapy at CCHE 57,357 during the study period, who met the inclusion criteria (actively engaged in chemotherapy-related clinical practices, regardless of qualifications or age, and willing to participate), were identified as eligible (*N* = 81). All 81 eligible nurses were approached, all agreed to participate and provided consent, and all 81 completed both the pre- and post-intervention assessments of knowledge and practice regarding mucormycosis prevention, with no exclusions or losses to follow-up.

### Sample size

The total eligible population of nurses actively involved in the care of children receiving chemotherapy at CCHE 57,357 was 81. Therefore, a total population sampling (census) approach was adopted, and all eligible, consenting nurses were enrolled. As supporting justification, an a priori power calculation was performed using G*Power for a paired-samples design, assuming a medium effect size (dz = 0.5), a two-tailed α = 0.05, a power of 95%, and an assumed pre-post correlation of *r* = 0.5 in the absence of prior empirical data on this specific instrument. This yielded a minimum required sample of approximately 54 participants, which was less than the total eligible population. Therefore, all 81 eligible nurses were enrolled using a census approach. No participants were excluded or lost to follow-up.

**Figure:**
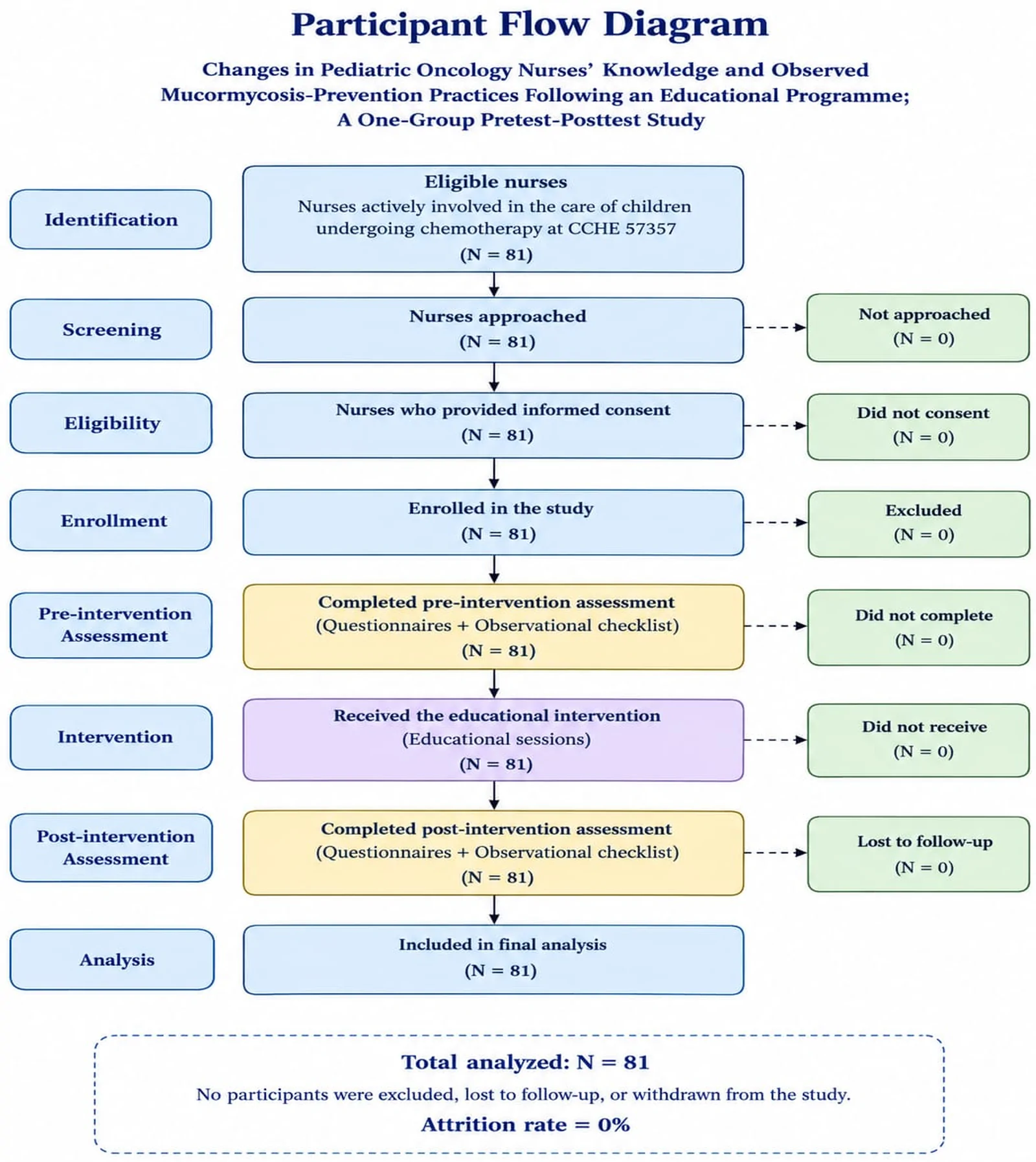

### Data collection measures

A structured questionnaire was developed to collect the data, consisting of three sections, and it is provided as a supplementary file:

#### Part I: Sociodemographic and work-related characteristics

This part collected data on participants’ sociodemographic characteristics and work-related information, including age, gender, qualification, years of nursing experience, and previous training related to mucormycosis prevention.

#### Part II: nurses’ knowledge assessment

The knowledge assessment tool was developed by the researchers following a comprehensive review of the relevant literature and the Global Guidelines for the Diagnosis and Management of Mucormycosis. The tool consisted of 15 items covering the nurses’ knowledge of mucormycosis, including risk factors, causes, clinical manifestations, prevention, complications, and management. Each completely correct answer was assigned a score of 1, whereas an incorrect answer or “I do not know” response was assigned a score of 0.

#### Part III: observational checklist of nurses’ practices

An observational checklist consisting of 9 items was used to assess nurses’ practices regarding mucormycosis prevention. The checklist items were developed based on relevant recommendations from the Centers for Disease Control and Prevention (CDC) and adapted to the pediatric oncology setting [21]. Each correctly performed practice was scored 1, whereas a practice that was not performed was scored 0. Observations were conducted by a single trained member of the research team, who observed each nurse once during the pre-intervention period and once during the post-intervention period while she performed her routine clinical duties. Because a single observer carried out all assessments, inter-rater reliability could not be calculated; this is noted as a limitation.

Total scores were calculated by summing the knowledge score (0–15) and the practice score (0–9) into a combined score out of 24, then converted to a percentage. Based on this combined percentage, overall performance was categorized as good (> 80%; 20–24 points), fair (60–80%; 15–19 points), or poor (< 60%; <15 points), based on Bloom’s cut-off point [22].

### Validity and reliability

Validity and reliability: The content validity of the knowledge questionnaire and observational practice checklist was assessed by five experts in pediatric nursing. The item-level content validity index (I-CVI) was calculated as the proportion of experts rating each item as either 3 or 4 on the relevance scale. For the 15-item knowledge questionnaire, the I-CVI ranged from 0.80 to 1.00, with a scale-level content validity index based on the average method (S-CVI/Ave) of 0.93. For the nine-item observational practice checklist, the I-CVI ranged from 0.80 to 1.00, with an S-CVI/Ave of 0.91.

Internal consistency of the binary knowledge questionnaire was assessed using the Kuder–Richardson 20 (KR-20), which yielded a coefficient of 0.768. The internal consistency of the observational practice checklist was assessed using Cronbach’s alpha, with a coefficient of 0.761.

### Pilot study

A pilot study was conducted on 10% of the total eligible sample (8 nurses) to assess the clarity, comprehensibility, applicability, and feasibility of the study tools and data collection procedures. Based on the results of the pilot study, no modifications or refinements were required, and the pilot participants were included in the main study sample.

### Intervention

The intervention consisted of structured educational sessions designed to enhance nurses’ knowledge and practices concerning the prevention of mucormycotic infection, based on relevant recommendations from the Centers for Disease Control and Prevention (CDC) and adapted to the pediatric oncology setting [21]. The educational program was tailored to the nurses’ baseline knowledge and practice levels and was delivered in three sessions. The three sessions were delivered in small groups over a two-week period. The theoretical sessions were encompassing knowledge related to mucormycosis through two sessions first session included causes, epidemiology, and the risk factors that predispose children receiving chemotherapy to mucormycosis. The first session also covered the clinical manifestations of mucormycosis and the diagnostic methods used to confirm the disease. The second session covered the pathophysiology of mucormycosis and prevention strategies, offering an overview of measures to reduce infection risk in pediatric oncology settings, in addition to the potential complications of untreated mucormycosis, principles of antifungal management, and the importance of promptly reporting suspicious signs to the treating team. Each session lasted between 20 and 40 min and was delivered face-to-face in groups of 15 nurses, depending on their work schedules. Each nurse participated in three sessions of training. The sessions included case presentations, group discussions, and opportunities for participants to ask questions and provide feedback. The teaching strategies were designed to align with the content and objectives of each session, incorporating a variety of educational methods such as PowerPoint presentations, figures, videos, and reflective discussions. Also third session, (nurse’s practices) were carried out inside the hospital training room. The guidelines were explained to nurses in small groups (around fifteen nurses). Each nurse was informed about importance of hand hygiene, the proper use and disposal of personal protective equipment (PPE), environmental cleaning guidelines, and isolation precautions for suspected or confirmed cases of mucormycosis under the current study. The theoretical lectures and the practical demonstration sessions were delivered by two members of the research team holding academic ranks of Professor and Assistant Professor of Pediatric Nursing. Steps of hand hygiene, PPE and isolation precautions were explained with teaching materials and demonstrated by the researchers and re-demonstration were carried out by each nurse individually. The researchers observed each nurse, and reassured the nurse if any mistakes were committed, and discussed it took about 20 to 40 mints.

#### Evaluation of the intervention

Following the completion of the educational sessions, the nurses were re-evaluated to measure the impact of the intervention. This evaluation included a post-test questionnaire, which was administered to assess knowledge acquisition using the same 15-item questionnaire from the pre-test phase. Additionally, the observational checklist used during the pre-intervention phase was employed again to observe and score the nurses’ practices post-intervention. The post-test was administered immediately after the completion of the educational intervention to minimize recall decay and ensure that the measured outcomes reflected the direct effect of the intervention rather than external factors. After the post-test, an open discussion was held, allowing nurses to share their experiences and thoughts about the training, and ensuring that any lingering questions or concerns were addressed.

### Ethics approval and consent to participate

Ethical approval for this study was obtained from the Research Ethics Committee, Faculty of Nursing, October 6 University, Giza, Egypt (Approval No. REC-NU-71202610, dated January 7, 2026). Administrative permission to conduct the study was also obtained from the Children’s Cancer Hospital Egypt (57357) prior to data collection. All procedures performed in this study were conducted in accordance with the ethical standards of the institutional research committee and the Declaration of Helsinki and its later amendments. Written informed consent was obtained from all participants prior to their enrollment in the study.

### Statistical design

Data were analyzed using IBM SPSS Statistics, version 26. Descriptive statistics were used to summarize the study variables, including frequencies and percentages for categorical variables and means and standard deviations for continuous variables. The normality of the within-participant differences between pre-intervention and post-intervention knowledge and practice scores was assessed using the Shapiro–Wilk test and graphical inspection of Q–Q plots. The within-participant differences for both knowledge and practice scores were approximately normally distributed; therefore, paired-samples t-tests were used to compare pre-intervention and post-intervention scores. The mean paired change, standard deviation of the change, 95% confidence interval (CI) for the mean change, degrees of freedom (df), and two-sided p-values were reported. Paired effect sizes were calculated using Cohen’s d for paired samples. A p-value of <0.05 was considered statistically significant.

## Result 

### Validity and reliability of the study instruments

The content validity assessment demonstrated acceptable item-level content validity for both instruments. The I-CVI ranged from 0.80 to 1.00 for the knowledge questionnaire and observational practice checklist. The S-CVI/Ave was 0.93 for the knowledge questionnaire and 0.91 for the observational practice checklist. The 15-item knowledge questionnaire demonstrated acceptable internal consistency, with a KR-20 coefficient of 0.768, while the observational practice checklist demonstrated a Cronbach’s alpha coefficient of 0.761. The socio-demographic characteristics of the studied nurses are presented in Table 1. 

### Pre-and post-intervention analysis of preventive practices

The effectiveness of the educational intervention was evaluated by comparing the pre-and post-intervention practices of the nurses related to mucormycosis prevention. Table 2 details the distribution of the nurses’ preventive practices before and after the educational intervention.

### Comparison of pre-and post-intervention knowledge and practice scores

The impact of the educational intervention on the nurses’ knowledge and practices was further quantified by comparing the mean scores pre-and post-intervention. Table 3 presents the results of this analysis.

### Correlation between knowledge and practice

The correlation between the nurses’ knowledge and their practical application was analyzed both pre-and post-intervention. The results are summarized in Table 4.

## Discussion

### Background and context

Mucormycosis is the third most common fatal invasive fungal infection, posing a challenge and remaining a significant cause of morbidity and mortality, especially in immunocompromised children [23]. To the best of our knowledge, this is one of the few studies, and the first in Egypt, to evaluate changes following educational sessions on nurses for preventing mucormycotic infection among children receiving chemotherapy. Assessing and updating nurses’ knowledge and practices regarding mucormycosis prevention is crucial for reducing such infections among both nurses and hospitalized children. The present study hypothesized that nurses who participate in educational sessions to prevent mucormycotic infection among children receiving chemotherapy would improve their knowledge and practice compared to their prior intervention.

### Nurses’ knowledge and practice regarding the prevention of mucormycotic infection

In light of our findings, this study showed improvement in nurses’ knowledge regarding the prevention of mucormycotic infection among children receiving chemotherapy compared to preintervention scores, with statistically significant differences. Additionally, this study provides compelling evidence that nurses’ knowledge and practices improved following the educational sessions related to mucormycotic infection prevention in pediatric oncology settings. Moving forward, continued efforts to prioritize professional development and education are critical for ensuring the ongoing competence and preparedness of healthcare professionals in combating infectious diseases and safeguarding patient health.

This finding is consistent with Gohil and Deb [24], who observed improved knowledge post-intervention. Additionally, this result is supported by Budhagaonkar and Dhanawade [25], who found that most participants had poor knowledge about the prevention and management of mucormycosis as pre-intervention, highlighting the need for training programs. It also aligns with research by Koshy et al. [26], where significant increases in infection control knowledge were noted post-training. Furthermore, Oladele et al. [27] emphasize the critical need for continuous education to address knowledge gaps in invasive fungal infections, as seen in Nigerian healthcare settings. These gaps can be mitigated through improved and targeted continuing medical education programs [28]. While prior research focused primarily on knowledge as a standalone factor, our study contributes to the literature by demonstrating its practical impact through intervention-based improvements.

Concerning nurses’ preventive practices for mucormycosis, the findings of this study demonstrate a noteworthy improvement in nurses’ preventive practices for mucormycosis following targeted educational sessions. The statistically significant increase in the total nurses’ practice level from pre- to post-training sessions (*p* ≤ 0.001) is consistent with improved nurses’ preparedness and adherence to preventive measures following the intervention. The observed association between the training approach and improved nurses’ practices in the present study could be related to the use of pedagogical approaches that support nurses’ learning. Furthermore, the findings underscore the critical role of continuous training and education in maintaining high infection prevention and control standards.

The statistical results indicate a measurable improvement in knowledge and observed practices following the educational sessions compared with baseline assessments. These findings are consistent with the study objective of improving nurses’ awareness and application of infection prevention measures. The observed improvement in knowledge and practice scores provides evidence of enhanced performance following the intervention; however, the one-group pre–post design does not allow causal attribution of the observed changes solely to the educational sessions.

This finding was congruent with a study conducted by El Sebaey et al. [29], which reported that a training program is highly effective. All nurses should undergo infection control training to acquire the necessary knowledge and skills to combat the spread of infections in healthcare settings. The results of the present study are consistent with those of Shahin et al. [16], who reported highly statistically significant improvements in all preventive practices against mucormycosis after the interventions. They reported that the overall mean score for preventive practice assessment improved postintervention.

The observed improvements in both knowledge and practice among participating nurses underscore the importance of continuous professional development in the field of healthcare. Educating nurses with updated information and practical skills empowers them to deliver higher-quality care and mitigate the risk of infectious complications, thereby supporting patient safety and quality of care [30, 31].

### Correlation between nurses’ knowledge and practice

Our study investigated the intricate relationship between knowledge level and preventive practices, aiming to shed light on the effectiveness of intervention training approaches in promoting preventive behaviors. The findings revealed a noteworthy and highly statistically significant positive correlation between total knowledge and preventive practices both before and after the intervention training. These results have significant implications for public health interventions and underscore the importance of knowledge dissemination in fostering proactive health behaviors. The observed positive relationship between total knowledge and preventive practices aligns with the widely acknowledged theory of planned behavior [32], which posits that individuals are more likely to engage in preventive actions when they possess adequate knowledge about the associated risks and benefits.

From a public health perspective, these results emphasize the critical role of education and knowledge dissemination in driving behavioral change. By equipping individuals with essential knowledge, intervention programs can facilitate proactive health measures, thereby reducing the risk of adverse health outcomes. This insight reinforces the value of integrating educational components into public health strategies to foster long-term behavioral adjustments that support overall health.

This supports prior studies, including those by Atya et al. [33] and Soher et al. [34], which also found a positive correlation between knowledge and practice levels among healthcare workers post-training. By equipping nurses with updated knowledge, educational programs help bridge the knowledge-practice gap, ultimately enhancing adherence to infection control protocols and improving patient outcomes. Moreover, Khalaf et al. [35] reported a positive correlation between nurses’ knowledge of and practices related to the management of oral mucositis in children receiving oncology therapy.

### Factors affecting nurses’ practice

Regarding factors associated with nurses’ pre-intervention practice scores, age showed a statistically significant positive association with practice scores (B = 0.041, *p* = 0.034), whereas years of experience showed a positive but non-significant association (B = 0.028, *p* = 0.104). Knowledge score was also significantly and positively associated with pre-intervention practice scores (B = 0.184, *p* = 0.004), while qualification was not significantly associated with practice scores (B = 0.315, *p* = 0.195). Overall, the regression model was statistically significant (F(4,76) = 3.890, *p* = 0.012) and explained 17.0% of the variance in pre-intervention practice scores (R² = 0.170; adjusted R² = 0.126).

These results support the findings of the study by Aldawsari et al. [36], who reported that poor knowledge (adjusted OR = 2.575; 95% CI = 1.263, 5.246; *P* = 0.009) was a significant predictor of unsatisfactory practices among healthcare workers. Similarly, the study by Kabir et al. [37] found that mucormycosis perception scores were significantly associated with age.

### Limitations of study

Limitations of the study: This study has some limitations. The one-group pre–post design without a control group or randomization limits the ability to attribute the observed improvements solely to the educational intervention. The post-intervention assessment was conducted immediately after the sessions, so long-term retention and sustainability of the improvements could not be assessed. Knowledge assessment may have been affected by testing or recall effects, while direct observation of practice may have introduced observer or Hawthorne effects. The study was conducted in a single pediatric oncology setting with a relatively small sample, which may limit the generalizability of the findings. Actual mucormycosis incidence and patient-level outcomes were not measured, so any relationship between the observed practice changes and clinical infection rates remains unconfirmed. Because nurses were trained and assessed in groups, some contamination between participants (e.g., discussion of content between pre- and post-intervention assessments) cannot be ruled out. Practice was assessed by a single observer with one observation per nurse at each time point; consequently, inter-rater and intra-rater reliability could not be established, and single-observer assessment may be subject to unmeasured observer bias.

### Implication of practice

The implications of practice for this study highlight the importance of continuous infection control training for nurses, especially in pediatric and intensive care settings, to ensure high standards of patient care. Educational interventions and infection control programs have been shown to significantly improve nurses’ knowledge and practices, which in turn can reduce the transmission of infections and enhance patient outcomes. By implementing regular training and reinforcing infection control guidelines, healthcare facilities can empower nurses to adopt evidence-based practices, thus promoting a safer environment for vulnerable patients.

Strengthening these programs could also lead to a shift in the overall culture within healthcare settings, fostering a proactive approach to infection prevention and adherence to antimicrobial stewardship, as suggested by recent research. This emphasis on education and adherence to protocols is essential for mitigating infectious disease risks and improving patient care quality.

## Conclusion

Structured educational sessions were associated with significant short-term improvements in pediatric oncology nurses’ knowledge and observed mucormycosis-prevention practices. However, because the study used a single-group pretest-posttest design and did not assess patient outcomes or mucormycosis incidence, these findings should not be interpreted as evidence of reduced infection rates or improved clinical outcomes. Future research should incorporate follow-up assessments at 3 and 6 months to determine whether improvements in nurses’ knowledge and observed preventive practices are sustained over time.

**Table 1:** Characteristics of the studied nurses

Item	*N*	%
**Age**		
25 – <35	59	72.8
35 - <45	19	23.5
45–55	3	3.7
**Mean Age (SD)**	32.38 ± 4.8	
**Gender**		
Male	13	16
Female	68	84
**Qualification**		
Technical Health Nursing Degree	11	13.6
Bachelor’s Degree in Nursing	70	86.4
**Years of Experience**		
5 - <10	44	54.3
10 - <15	30	37.1
15–20	7	8.6
**Mean Years of Experience (SD)**	9.62 ± 2.9	
**Training Course on** **Mucormycosis Prevention**		
Yes	34	41.9
No	47	58.1

Table 1 provides a comprehensive summary of the demographic characteristics of the study participants, indicating a predominantly young, female nursing workforce with a strong educational background in nursing

**Table 2:** Preventive practices before and after the educational intervention (*N* = 81)

Preventive Practice	Pre *n* (%)	Post *n* (%)	(Not done → Done)	(Done → Not done)	Δ% (95% CI)	McNemar’s χ²	*P*-Value
**Educating children and caregivers on hand and respiratory hygiene**							
Done	10 (12.3%)	57 (70.4%)	48	1	58.0 (46.2–69.8)	43.31	< 0.001
Not done	71 (87.7%)	24 (29.6%)					
**Proper hand hygiene**							
Done	49 (60.5%)	78 (96.3%)	30	1	35.8 (23.8–47.8)	24.03	< 0.001
Not done	32 (39.5%)	3 (3.7%)					
**Wearing appropriate personal protective equipment (PPE)**							
Done	45 (55.6%)	77 (95.1%)	33	1	39.5 (27.8–51.2)	28.44	< 0.001
Not done	36 (44.4%)	4 (4.9%)					
**Closely monitoring children for signs and symptoms of mucormycosis.**							
Done	12 (14.8%)	72 (88.9%)	61	1	74.1 (63.4–84.8)	51.27	< 0.001
Not done	69 (85.2%)	9 (11.1%)					
**Assessing nutritional status of children receiving chemotherapy**							
Done	14 (17.3%)	69 (85.2%)	57	2	67.9 (56.6–79.2)	51.27	< 0.001
Not done	67 (82.7%)	12 (14.8%)					
**Wearing masks when visiting dusty sites**							
Done	15 (18.5%)	59 (72.8%)	46	2	54.3 (42.9–65.7)	42.09	< 0.001
Not done	66 (81.5%)	22 (27.2%)					
**Implementing and monitoring environmental cleaning and disinfection practices**							
Done	23 (28.4%)	69 (85.2%)	48	2	56.8 (45.0–68.6)	42.32	< 0.001
Not done	58 (71.6%)	12 (14.8%)					
**Wearing gloves during handling materials such as soil or moss**							
Done	46 (56.8%)	75 (92.6%)	30	1	35.8 (24.3–47.3)	25.48	< 0.001
Not done	35 (43.2%)	6 (7.4%)					
**Wearing shoes and long-sleeved shirts during outdoor activities**							
Done	27 (33.3%)	63 (77.8%)	38	2	44.4 (32.6–56.3)	32.40	< 0.001
Not done	54 (66.7%)	18 (22.2%)					

Table 2 demonstrates a statistically significant increase in all preventive practices following the intervention, with all p-values less than 0.001, suggesting a positive change in nurses’ preventive practices following the educational sessions. To account for multiple comparisons across the nine practice items, a Bonferroni-corrected significance threshold of α = 0.0056 (0.05/9) was applied; all nine McNemar tests remained statistically significant after this correction

**Table 3:** Changes in nurses’ knowledge and practice scores pre- and post-intervention (*N* = 81)

Measure	Pre-Intervention Mean ± SD	Post-Intervention Mean ± SD	Mean Change ± SD	95% CI of Change	t-test	df	*p*-value	Paired effect size (Cohen’s d)
*Total Knowledge*	*5.82 ± 0.91*	*12.10 ± 0.76*	***6.28 ± 0.84***	***6.09–6.47***	*67.198*	*80*	*< 0.001*	***7.47***
*Total Practice*	*3.86 ± 1.13*	*7.58 ± 1.023*	***3.72 ± 0.68***	***3.57–3.87***	*49.534*	*80*	*< 0.001*	***5.50***

Table 3 demonstrates significant increases in nurses’ knowledge and observed practice scores following the educational sessions. The mean knowledge score increased from 5.82 ± 0.91 at pre-intervention assessment to 12.10 ± 0.76 at post-intervention assessment, while the mean practice score increased from 3.86 ± 1.13 to 7.58 ± 1.02 (p < 0.001). These findings indicate significant post-intervention changes in knowledge and observed preventive practices. However, given the one-group pretest-posttest design and the immediate post-intervention assessment, these changes cannot be attributed solely to the educational sessions. The within-participant differences in knowledge and practice scores were approximately normally distributed, supporting the use of paired-samples t-tests

**Table 4:** Correlation between total knowledge and practice scores pre- and post-intervention

Variables	*r*.	*p*.
Total knowledge & practice pre	0.348	0.001
Total knowledge & practice post	0.382	< 0.001

As indicated in Table 4, there was a positive and statistically significant correlation between knowledge and practice both before intervention (*r* = 0.348, *p* = 0.001) and after intervention (*r* = 0.382, *p* < 0.001). Although the post-intervention correlation coefficient was slightly higher, the magnitude of both correlations reflects a weak-to-moderate association, and the difference between the two coefficients was not formally tested for statistical significance. Therefore, this finding should be interpreted with caution rather than as clear evidence of a strengthened relationship between knowledge and practice following the intervention

A multiple linear regression analysis was performed to identify factors associated with nurses’ pre-intervention practice scores

The results of the regression analysis are presented in Table 5

**Table 5:** Multiple linear regression analysis of factors associated with nurses’ pre-intervention practice scores

Predictor	B	SE	β	95%C1 for B	t	*p*-value	Tolerance	VIF
Age	0.041	0.019	0.231	**0.003–0.079**	**2.158**	**0.034**	**0.742**	**1.348**
Years of experience	0.028	0.017	0.182	-0.006-0.062	1.647	0.104	0.721	1.387
Qualification	0.315	0.241	0.119	-0.165-0.795	1.307	0.195	0.918	1.089
Knowledge score	0.184	0.061	0.315	0.063–0.305	2.994	0.004	0.803	1.245
**Model: F**(4,76) = 3.890, p = 0.012, **R²**= 0.170, **Adjusted R²**= 0.126; **Durbin-Watson = 1.87. Residual diagnosis: Shapiro-Wilk test**,** p** = 0.126								

Table 5 shows that the multiple linear regression model was statistically significant (F(4,76) = 3.890, p = 0.012), explaining 17.0% of the variance in nurses’ pre-intervention practice scores (R² = 0.170; adjusted R² = 0.126). Knowledge score was positively and significantly associated with pre-intervention practice scores (B = 0.184, β = 0.315, t = 2.994, p = 0.004). Age was also positively associated with practice scores and was statistically significant (B = 0.041, β = 0.231, t = 2.158, p = 0.034). Years of experience showed a positive but non-significant association with practice scores (B = 0.028, β = 0.182, t = 1.647, p = 0.104), while qualification was not significantly associated with practice scores (B = 0.315, β = 0.119, t = 1.307, p = 0.195). The tolerance and VIF values did not indicate substantial multicollinearity among the predictors. The Shapiro–Wilk test and Durbin–Watson statistic did not indicate major concerns regarding the normality of residuals and independence of errors, respectively

## Data Availability

The datasets used and/or analyzed during the current study are available from the corresponding author on reasonable request.

## Abbreviations

CCHE: Children's cancer hospital Egypt
LIFE: Leading international fungal education
HMs: Hematologic malignancies
HSCT: Hematopoietic stem cell transplant
CDC: Centers for disease control and prevention
PPE: Personal protective equipment
